# Cyclin-dependent kinase-specific activity predicts the prognosis of stage I and stage II non-small cell lung cancer

**DOI:** 10.1186/1471-2407-14-755

**Published:** 2014-10-09

**Authors:** Hiroshi Kubo, Takashi Suzuki, Tomoko Matsushima, Hideki Ishihara, Kazuya Uchino, Satoshi Suzuki, Sachiyo Tada, Masahiro Yoshimura, Takashi Kondo

**Affiliations:** Department of Advanced Preventive Medicine for Infectious Disease, Tohoku University Graduate School of Medicine, 2-1 Seiryoumachi, Aobaku, Sendai, 980-8575 Japan; Department of Pathology and Histotechnology, Tohoku University Graduate School of Medicine, 2-1 Seiryoumachi, Aobaku, Sendai, 980-8575 Japan; Central Research Laboratories, Sysmex Corporation, 4-4-4, Takatsukadai, Nishi-ku, Kobe, 651-2271 Japan; Department of General Thoracic Surgery, Hyogo Cancer Centre, 13-70 Kitaouji-chou, Akashi, 673-8558 Japan; Department of Thoracic Surgery, Japanese Red Cross Ishinomaki Hospital, 71 Nishimichishita, Hebita, Ishinomaki, 986-8522 Japan; Department of Thoracic Surgery, Institute of Development, Aging and Cancer, Tohoku University, 4-1 Seiryoumachi, Aobaku, Sendai, 980-8575 Japan; R&D Department, Nittobo Medical Co. Ltd, 1 Shiojima Fukuhara, Fukuyama, Koriyama, 963-8061 Japan

**Keywords:** Non-small cell lung cancer, Cyclin-dependent kinase, Surgical resection, Recurrence, Mortality

## Abstract

**Background:**

Lung cancer is one of the leading causes of cancer death worldwide. Even with complete resection, the prognosis of early-stage non-small cell lung cancer is poor due to local and distant recurrence, and it remains unclear which biomarkers are clinically useful for predicting recurrence or for determining the efficacy of chemotherapy. Recently, several lines of evidence have indicated that the enzymatic activity of cyclin-dependent kinases could be a clinically relevant prognostic marker for some cancers. We investigated whether the specific activity of cyclin-dependent kinases 1 and 2 could predict recurrence or death in early non-small cell lung cancer patients.

**Methods:**

Patients with newly diagnosed, pathologically confirmed non-small cell lung cancer were entered into this blinded cohort study. The activity of cyclin-dependent kinases was determined in 171 samples by the C2P® assay, and the results were subjected to statistical analysis with recurrence or death as a clinical outcome.

**Results:**

The Cox proportional hazards model revealed that the activity of cyclin-dependent kinase 1, but not 2, was a predictor of recurrence, independent of sex, age, and stage. By contrast, cyclin-dependent kinase 2 activity was a predictor of death, independent of sex and stage.

**Conclusion:**

This study suggested the possible clinical use of cyclin-dependent kinase 1 as a predictor of recurrence and cyclin-dependent kinase 2 as a predictor of overall survival in early-stage non-small cell lung cancer. Thus, a combination of activity of cyclin-dependent kinases 1 and 2 is useful in decision-making regarding treatment strategies for non-small cell lung cancer after surgery.

**Electronic supplementary material:**

The online version of this article (doi:10.1186/1471-2407-14-755) contains supplementary material, which is available to authorized users.

## Background

Lung cancer is one of the leading causes of cancer death worldwide. Despite recent advances in cancer treatment, the prognosis of lung cancer is not sufficient compared with that of other solid organ tumors [1]. Even after complete surgical resection, the 5-year survival of early-stage non-small cell lung cancer (NSCLC) patients is only approximately 65% [2–4]. This poor prognosis is due to the high recurrence after resection [5, 6], which supports the presence of occult metastases. The survival benefit of adjuvant platinum-based chemotherapy has been established in stage II–III NSCLC [7–9]; however, there is no data supporting the use of adjuvant treatments for stage IA NSCLC, and the use of adjuvant chemotherapy for stage IB NSCLC is controversial [10]. Recently developed molecular biomarkers predict only non-squamous NSCLC [11, 12], and no biomarkers for squamous cell carcinoma (SCC) have reached the validation stage. Therefore, in the setting of lung cancer, the identification of biomarkers for predicting the outcome after surgery and selecting patients who could benefit from adjuvant chemotherapy is crucial.

A breakdown in the cell cycle machinery induces the uncontrolled proliferation of tumors. This process is initiated by a variety of molecules in a cascade that activates the cyclin-dependent kinases (CDKs), which play a role in the progression of the cell cycle. On the molecular level, the activity of CDKs is regulated by subunits known as cyclins, and by phosphorylation and dephosphorylation of key residues, for example, Thr14, Tyr15, and Thr160 in CDK2 [13]. A series of pathological investigations of the molecules that stimulate CDKs have clearly demonstrated their clinical significance for cancer diagnosis and treatment. For example, clinical evidence has indicated that the overexpression of cyclin E and cyclin B, which bind to and activate CDK2 and CDK1, respectively, correlates with tumorigenesis, prognosis, and sensitivity to chemotherapy in a variety of malignancies, as does the inactivation of CDK inhibitors, such as p21WAF1 and p27Kip1 [14–20]. The pairing between the CDK and cyclin isotypes is specific. However, the amount of cyclin protein did not correspond perfectly with CDK activity in our investigation (data not shown). Similar results regarding the association between cyclin E and the activity of its associated kinase were reported by another group [21]. Therefore, we hypothesized that the direct measurement of CDK activity might produce relevant clinical indications for cancer diagnosis and treatment. Previously, we reported that the CDK-based risk score (C2P® assay, Sysmex, Japan) predicted the risk of distant recurrence in early breast cancer patients [22]. The C2P® assay is determined using a combination of the specific activity of CDK1 (CDK1SA) and CDK2 (CDK2SA). The feasibility of this assay was confirmed in a cohort study in Caucasian breast cancer patients [23] and in colon cancer patients [24]. CDKSAs were also significantly associated with a pathologically complete response (pCR) after weekly administration of paclitaxel followed by 5-fluorouracil, epirubicin, and cyclophosphamide in breast cancer [25].

In lung cancer, many cell cycle-related molecules have been reported to be correlated with prognosis [26–31]. Here, we investigated whether the activity of CDK1 and CDK2 could predict the recurrence of NSCLC or the death of stage I and II NSCLC patients.

## Methods

### Study design

This blinded cohort study was approved by the local ethics committees of Tohoku University and Hyogo Cancer Centre. All patients provided written informed consent. A total of 213 patients who were newly diagnosed with pathologically confirmed NSCLC at the two centers were enrolled in this study. The eligibility criteria were as follows: SCC, adenocarcinoma, and stage I–II disease. All patients underwent complete resection, and none received adjuvant or neoadjuvant chemotherapy. CDK1SA and CDK2SA were determined in 171 samples using a C2P® assay (Sysmex, Kobe, Japan), and the results were subjected to statistical analysis to evaluate recurrence or death as a clinical outcome. Tumor tissue was dissected immediately after resection, snap-frozen and stored at -80°C at each facility. Then, the samples were sent to the Sysmex Corporation (Kobe, Japan) and subjected to the C2P® assay. Tissues with extreme blood contamination were excluded from this study, because the expression level of CDKs is underestimated in the presence of more than 1600 ng/μL of hemoglobin. The histologic types were centrally reviewed at Tohoku University.

### Patients

A total of 213 patients with primary NSCLC who had undergone surgery between July 2000 and September 2009 were recruited for this study. Twenty-four cases were excluded due to extreme blood contamination of the samples. C2P® assay measurements were performed on 189 frozen samples; in 18 cases, the CDK expression levels were below the detection threshold, and these samples were excluded from the analysis. Finally, 171 cases were subjected to statistical analyses, including 53 SCCs and 118 adenocarcinomas. The median follow-up period was 43.9 months (70–2820 days).

### Measurement of CDK1SA and CDK2SA

The C2P® assay [15, 22] was used to measure the specific activity of CDKs. In brief, lysates of freshly frozen samples were applied to the wells of a 96-well PVDF filter plate (Millipore, Billerica, MD, USA). The expression of CDK protein was detected quantitatively by sequential reactions with primary anti-CDK antibodies, biotinylated anti-rabbit antibodies and fluorescein-labeled streptavidin. To measure the kinase activity, CDK molecules were immunoprecipitated from the lysate using protein beads. CDK SA activity (maU/eU) was calculated as CDK activity units (maU/μL lysate), which were divided by their corresponding CDK expression units (eU/μL lysate). maU (CDK activity unit) and eU (CDK expression unit) were defined as the enzyme activity and expression equivalent to 1 ng of recombinant kinase, respectively. When the expression level was lower than the detection limit of the assay, the case was excluded from the analysis. The detection limits for the expression level of CDK1 and CDK2 are 0.1 and 0.003 eU/μL lysate, respectively.

### Statistical analysis

Recurrence-free survival (RFS) was calculated from the date of surgery to the date of first local or distant recurrence; patients who were alive without recurrence at the time of data collection and those who died without any evidence of the disease on the date of death were censored. The overall survival (OS) was calculated from the date of surgery to the date of death; patients who were alive were censored.

The data were analyzed using MedCalc version 12.3 (MedCalc Software, Ostend, Belgium), and survival between the groups was compared using the Kaplan-Meier method and an unstratified Cox proportional hazards model or the log-rank test. Correlation tables were analyzed using the chi-square test. Receiver operating characteristic (ROC) curves and the corresponding area under the curve (AUC) for the compared models were computed to simulate predictive accuracy.

Possible prognostic variables that were analyzed in this study included age (≥70 *vs* <70 years), sex (male or female), tumor size (>3 *vs* ≤3 cm), nodal status (negative or positive), pathological stage (≥IB *vs* IA), histological type (SCC or adenocarcinoma), CDK1SA (≥12.6 *vs* <12.6) and CDK2SA (≥222 *vs* <222). A value of *p* <0.05 was considered significant.

## Results

**Figure 1:**
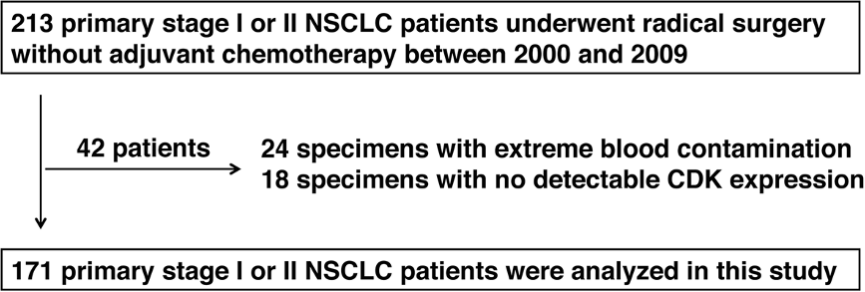
**Flow chart of patient enrolment and reasons for exclusion.**

We obtained fresh-frozen samples from 213 cases from two centers: Tohoku University Hospital and Hyogo Cancer Centre. Twenty-four cases were excluded due to extreme blood contamination. The C2P® assay was performed on 189 frozen samples (see Additional file 1); in 18 cases, the CDK expression levels were below the detection threshold, and these cases were excluded from the analysis (assay success rate =90%). Finally, 171 samples were subjected to statistical analysis (Figure 1). The patients who were analyzed included 106 (62%) males and 65 (38%) females, with a median age of 70 years (38–86) (Table 1). The median tumor size was 3.0 cm (0.9–10.0). A total of 150 cases (88%) were node-negative, and 21 cases (12%) were positive. The histologic type was centrally confirmed as SCC in 53 cases (31%) and adenocarcinoma in 118 cases (69%). The overall recurrence rate and overall survival rate at final follow-up were 22% (local, 8%; distant, 14%) and 78% (133/171), respectively. Thirty-five out of 37 recurrent cases received platinum-based chemotherapy. The distribution of CDK1SA and CDK2SA did not vary significantly between the two independent cohorts based on the chi-square test (*p* =0.2102 and *p* =0.3557, respectively; Table 2). To examine the prognostic significance of the CDK1SA and CDK2SA results, ROC analysis was performed with overall recurrence as a clinical outcome.

**Table 1:** **Clinical and pathological characteristics of patients**

		Total collective	Tohoku University	Hyogo Cancer Centre
Frozen blocks available		213	111	102
Successful CDK assay		171	90	81
Sex	Male	106 (62%)	57 (63%)	49 (60%)
	Female	65 (38%)	33 (37%)	32 (40%)
Age	< 70 years	83 (49%)	43 (48%)	40 (49%)
	≥ 70 years	88 (52%)	47 (52%)	41 (51%)
Tumor size	≤ 3 cm	95 (56%)	52 (58%)	43 (53%)
	> 3 cm	76 (44%)	38 (42%)	38 (47%)
pN	-	150 (88%)	83 (92%)	67 (83%)
	+	21 (12%)	7 (8%)	14 (17%)
Stage	IA	89 (52%)	48 (53%)	41 (51%)
	IB	45 (26%)	28 (31%)	17 (21%)
	IIA	31 (18%)	11 (12%)	20 (25%)
	IIB	6 (4%)	3 (3%)	3 (4%)
Histology	Adenocarcinoma	118 (69%)	58 (64%)	60 (74%)
	SCC	53 (31%)	32 (36%)	21 (26%)
Recurrence	None	134 (78%)	81 (90%)	53 (65%)
	Local	14 (8%)	3 (3%)	11 (14%)
	Distant	23 (14%)	6 (7%)	17 (21%)
Survival information	Alive	133 (78%)	75 (83%)	58 (72%)
	Dead	38 (22%)	15 (17%)	23 (28%)

**Table 2:** **Association between CDK-based risk groups and clinicopathological parameters**

		CDK1SA (cut-off = 12.6)			CDK2SA (cut-off = 222)		
		Low	High	Significance (Chi-square)	Low	High	Significance (Chi-square)
Sex	Male	57	49		76	30	
	Female	35	30	*p* =0.8817	55	10	*p =*0.0800
Age	< 70 years	43	40		66	17	
	≥ 70 years	49	39	*p =*0.7230	65	23	*p =*0.4888
Tumor size	≤ 3 cm	55	40		76	19	
	> 3 cm	37	39	*p =*0.2955	55	21	*p =*0.3223
pN	-	85	65		118	32	
	+	7	14	*p =*0.0759	13	8	*p =*0.1544
Stage	IA	53	36		73	16	
	IB	25	20		37	8	
	IIA	12	19		18	13	
	IIB	2	4	*p =*0.1652	3	3	*p =*0.0267
Histology	Adenocarcinoma	64	54		101	17	
	SCC	28	25	*p =*0.9961	30	23	*p* <0.0001
Facility	Tohoku Univ	53	37		72	18	
	Hyogo CC	39	42	*p =*0.2102	59	22	*p =*0.3557

The area under the ROC curves (AUC) of CDK1SA (*p* =0.0498) and CDK2SA (*p* =0.4206) were 0.607 and 0.545, respectively, which indicated that CDK1SA, but not CDK2SA, was likely to be predictive of recurrence. The analysis revealed that the Youden Indexes for CDK1SA and CDK2SA were maximized at 12.6 maU/eU and 222 maU/eU, respectively. Therefore, these values were tentatively set as the cut-off points in this study. Coincidentally, the optimal cut-off value of lung cancer approximated that of our colon study (11 maU/eU) [24]. The correlation analyses between CDKSA and the clinicopathologic parameters revealed that none of the parameters was associated with CDK1SA, while CDK2SA was significantly correlated with stage (*p* =0.0267) and histology (*p* <0.0001) (Table 2).

**Figure 2:**
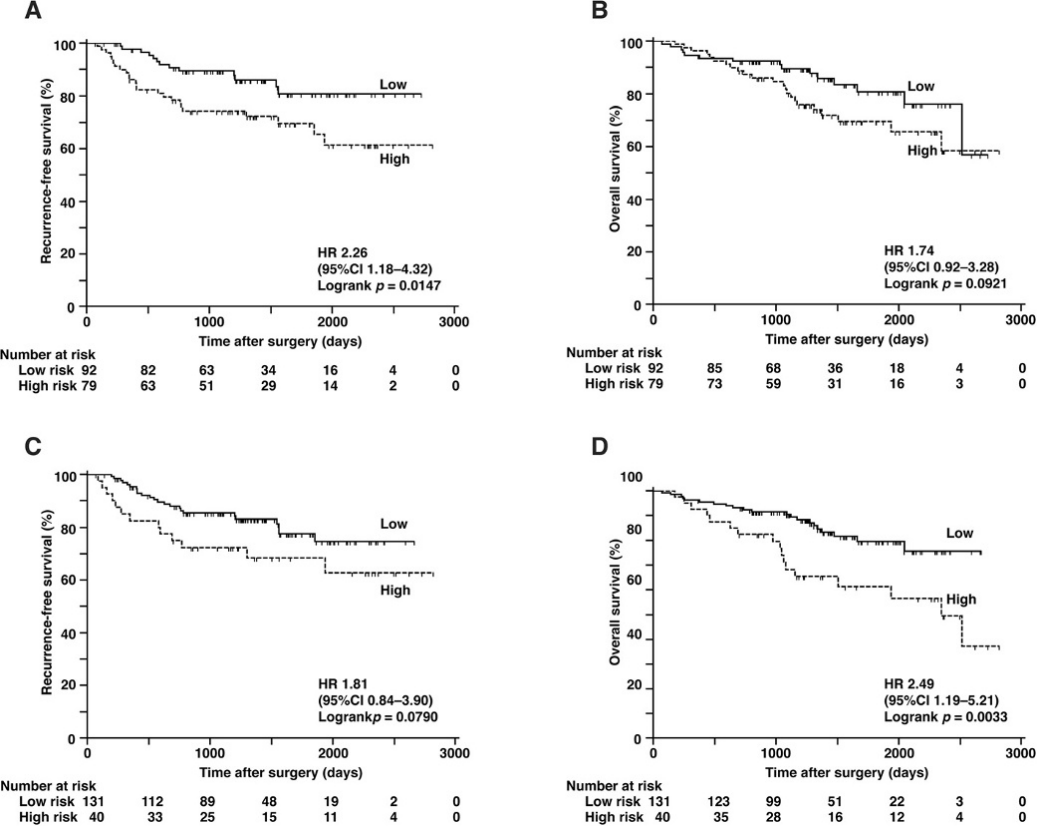
**Analysis of recurrence and survival by risk category. (A)** Recurrence-free survival and **(B)** overall survival according to CDK1SA-based risk with a cut-off value of 12.6 maU/eU. **(C)** Recurrence- free survival and **(D)** overall survival according to CDK2SA-based risk with a cut-off value of 222 maU/eU.

With a cut-off value of 12.6 maU/eU, the cases were classified as low CDK1SA (54%, 92 cases) or high CDK1SA (46%, 79 cases). In the Kaplan-Meier analysis, patients with low CDK1SA tumors showed significantly higher RFS than those with high CDK1SA tumors based on a log-rank test (Figure 2A, HR 2.26, 95% CI 1.18–4.32; *p* =0.0147); however, no prognostic value was observed (Figure 2B, *p* =0.0921).

**Figure 3:**
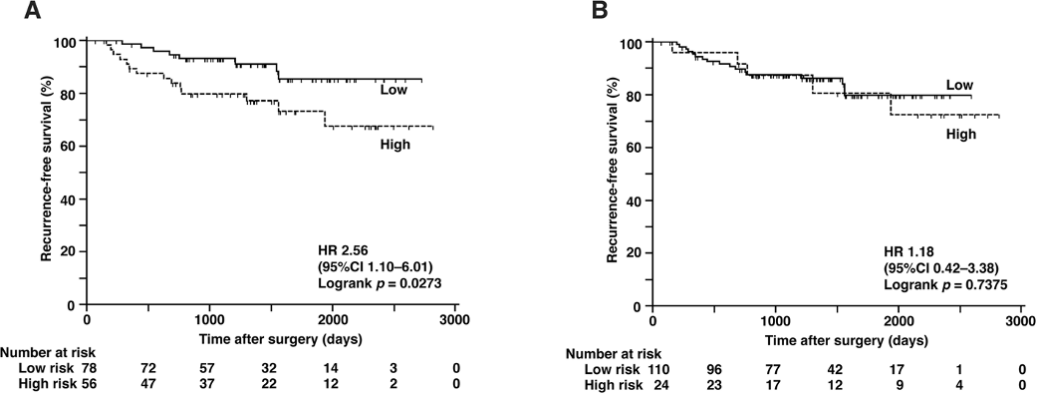
**Analysis of recurrence by risk category in stage I NSCLC. (A)** Recurrence-free survival according to CDK1SA-based risk with a cut-off value of 12.6 maU/eU. **(B)** Recurrence-free survival according to CDK2SA-based risk with a cut-off value of 222 maU/eU.

CDK1SA and conventional clinicopathologic parameters, including sex (male *vs* female), age (<70 *vs* ≥70), tumor size (≤3 cm *vs* >3 cm), pathological lymph node status (positive *vs* negative), clinical stage (IA *vs* IB-IIB), and histology (SCC *vs* adenocarcinoma) were analyzed using a Cox proportional hazards model with recurrence or death as a clinical outcome (Tables 3 and 4). Univariate analysis for recurrence revealed that sex (HR 2.53, 95% CI 1.16–5.52; *p* =0.0200), age (HR 2.80, 95% CI 1.36–5.77; *p* =0.0054), tumor size (HR 1.99, 95% CI 1.04–3.81; *p* =0.0380), pathological lymph node status (HR 3.20, 95% CI 1.51–6.77; *p* =0.0025), stage (HR 2.54, 95% CI 1.30–4.99; *p* =0.0070) and CDK1SA (HR 2.26, 95% CI 1.16–4.43; *p* =0.0177) were statistically significant (*p* <0.05). Age, stage and CDK1SA remained significant by multivariate analysis for recurrence (Table 3, age HR 3.06, *p* =0.0028; stage HR 2.16, *p* =0.0306; CDK1SA HR 2.25, *p* =0.0195). Even in the subgroup of 134 patients with stage IA and IB disease, CDK1SA but not CDK2SA had the prognostic power (Figures 3A and B, CDK1SA HR 2.56, *p* =0.0273; CDK2SA HR 1.18, *p* =0.7375). The Cox regression analysis revealed that CDK1SA was an independent predictor of recurrence (Table 5, HR 2.57, 95% CI 1.08–6.09; *p* =0.0335).

**Table 3:** **Cox proportional hazards models for recurrence**

		Univariate analysis		Multivariate analysis		Multivariate analysis	
		HR (95% CI)	***p***value	HR (95% CI)	***p***value	HR (95% CI)	***p***value
Sex	Male	2.53 (1.16–5.52)	0.0200	1.99 (0.90–4.42)	0.0913	2.00 (0.91–4.40)	0.0876
Age	≥ 70 years	2.80 (1.36–5.77)	0.0054	3.06 (1.48–6.34)	0.0028	2.79 (1.35–5.75)	0.0056
Tumor size	> 3 cm	1.99 (1.04–3.81)	0.0380				
pN	+	3.20 (1.51–6.77)	0.0025				
Stage	≥ IB	2.54 (1.30–4.99)	0.0070	2.16 (1.08–4.31)	0.0306	2.23 (1.13–4.43)	0.0222
Histology	SCC	1.23 (0.62–2.44)	0.5567				
CDK1SA	≥ 12.6	2.26 (1.16–4.43)	0.0177	2.25 (1.14–4.42)	0.0195		
CDK2SA	≥ 222	1.82 (0.93–3.56)	0.0837			1.53 (0.79–3.01)	0.2165

**Table 4:** **Cox proportional hazards models for death**

		Univariate analysis		Multivariate analysis		Multivariate analysis	
		HR (95% CI)	***p***value	HR (95% CI)	***p***value	HR (95% CI)	***p***value
Sex	Male	5.51 (1.96–15.46)	0.0013	4.29 (1.50–12.30)	0.0070	4.14 (1.45–11.78)	0.0081
Age	≥ 70 years	1.24 (0.66–2.35)	0.5088				
Tumor size	> 3 cm	2.36 (1.22–4.55)	0.0111				
pN	+	2.82 (1.34–5.96)	0.0069				
Stage	≥ IB	2.75 (1.39–5.44)	0.0039	1.79 (0.87–3.71)	0.1174	2.09 (1.03–4.24)	0.0421
Histology	SCC	2.49 (1.31–4.72)	0.0053	1.53 (0.78–2.97)	0.2156	1.23 (0.62–2.47)	0.5555
CDK1SA	≥ 12.6	1.74 (0.91–3.32)	0.0962	1.53 (0.79–2.97)	0.2122		
CDK2SA	≥ 222	2.56 (1.34–4.87)	0.0045			1.97 (1.00–3.87)	0.0500

**Table 5:** **Cox proportional hazards models for recurrence (Stage I)**

		Univariate analysis	
		HR (95% CI)	***p***value
Sex	Male	2.17 (0.85–5.53)	0.1050
Age	≥ 70 years	1.79 (0.75–4.24)	0.1908
Tumor size	> 3 cm	1.41 (0.59–3.35)	0.4413
Stage	≥ IB	1.67 (0.71–3.90)	0.2390
Histology	SCC	1.10 (0.41–2.96)	0.8568
CDK1SA	≥ 12.6	2.57 (1.08–6.09)	0.0335
CDK2SA	≥ 222	1.19 (0.44–3.21)	0.7381

Regarding CDK2SA, which had a cut-off value of 222 maU/eU, the cases were classified as low CDK2SA (77%, 131 cases) or high CDK2SA (23%, 40 cases). Patients with low CDK2SA tumors showed significantly higher OS than those with high CDK2SA tumors based on a log-rank test (Figure 2D, HR 2.49, 95% CI 1.19–5.21; *p* =0.0033).

According to the univariate analysis for death, CDK2SA (HR 2.56, 95% CI 1.34–4.87; *p* =0.0045) was statistically significant along with sex (HR 5.51, 95% CI 1.96–15.5; *p* =0.0013), tumor size (HR 2.36, 95% CI 1.22–4.55, *p* =0.0111), pathological lymph node status (HR 2.82, 95% CI 1.34–5.96; *p* =0.0069) and stage (HR 2.75, 95% CI 1.39–5.44; *p* =0.0039). By multivariate analysis, sex, stage, and CDK2SA remained significant (sex HR 4.14, *p* =0.0081; stage HR 2.09, *p* =0.0421; CDK2SA HR 1.97, *p* =0.0500).

**Figure 4:**
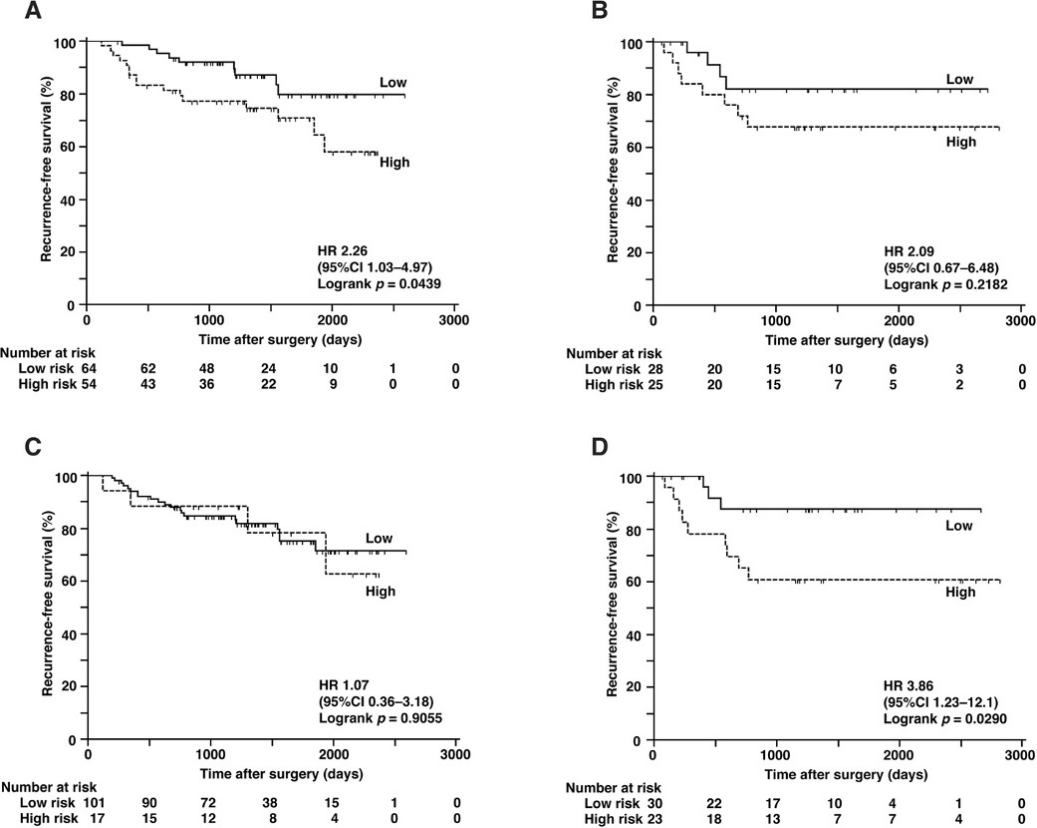
**Analysis of recurrence by risk category in adenocarcinoma (A) (C) and SCC (B) (D). (A)(B)** Recurrence-free survival according to CDK1SA-based risk with a cut-off value of 12.6 maU/eU. **(C)(D)** Recurrence-free survival according to CDK2SA-based risk with a cut-off value of 222 maU/eU.

Subanalysis by histology revealed that the predictive value of CDK1SA for recurrence was stronger in adenocarcinoma than in SCC (Figure 4A and B, adenocarcinoma HR 2.26, *p* =0.0439; SCC HR 2.09, *p* =0.2182). On the contrary, in the SCC cases, the Cox regression analysis for recurrence revealed that only CDK2SA was statistically significant (Figure 4C and D, Table 6, HR 3.86, 95% CI 1.05–14.2; *p* =0.0428).

**Table 6:** **Cox proportional hazards models for recurrence (SCC)**

		Univariate analysis	
		HR (95% CI)	***p***value
Sex	Male	NA	
Age	≥70 years	1.48 (0.47–4.65)	0.5014
Tumor size	>3 cm	1.77 (0.48–6.52)	0.3905
pN	+	1.94 (0.59–6.44)	0.2793
Stage	≥IB	2.42 (0.54–10.99)	0.2530
CDK1SA	≥12.6	2.09 (0.63–6.91)	0.2286
CDK2SA	≥222	3.86 (1.05–14.17)	0.0428

NA, not analyzed due to bias (89% of SCC patients were male).

**Figure 5:**
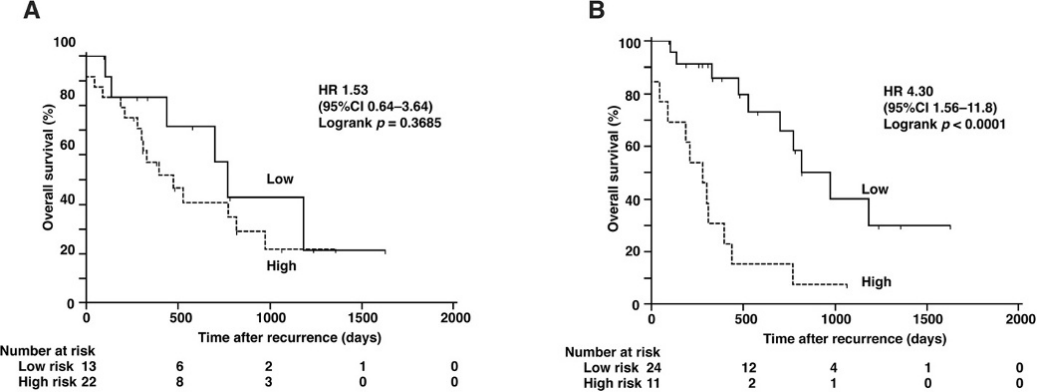
**Analysis of survival by risk category in recurrent cases.** The zero timepoint indicates the diagnosis of recurrence. **(A)** Survival according to CDK1SA-based risk with a cut-off value of 12.6 maU/eU. **(B)** Survival according to CDK2SA-based risk with a cut-off value of 222 maU/eU.

In the distribution of 35 recurrent cases on a scatter diagram with logarithmic scales according to CDK1SA and CDK2SA, we observed that the distribution of the non-survivors slightly shifted to the higher CDKSAs area (data not shown). This observation let us to perform Kaplan-Meier analyses, and it was found that the prognostic power of CDK2SA, but not of CDK1SA (Figure 5A), was significant in 35 patients who were treated with chemotherapy after recurrence (Figure 5B, HR for death 4.30, 95% CI 1.56–11.8; *p* <0.0001).

## Discussion

In this study, we demonstrated that CDK1SA and CDK2SA could identify individuals that were at high risk for recurrence and death among early-stage NSCLC patients after surgical resection. Even with complete resection, the prognosis of early-stage NSCLC is not good due to local and distant recurrence [6], and it remains unclear which biomarkers are clinically useful for predicting recurrence, although some single molecules and gene signatures of non-squamous cell carcinomas are being validated in a large number of cohorts [11, 32]. Previously, cyclin expression and prognosis were reported to be correlated in lung cancer patients [33]; however, this is the first study revealing that CDK activity is a promising predictor for early-stage NSCLCs. Furthermore, CDK1SA and CDK2SA are the first biomarkers that can be used to predict the prognosis of both adenocarcinoma and SCC. Because CDKs are the targets of new anti-cancer drugs and the development of many CDK inhibitors is underway [34], this study will provide a basis for future personalized medicine using CDK inhibitors in NSCLC patients.

CDK1SA and CDK2SA have different implications in lung cancer. CDK1SA predicted recurrence after surgery, whereas CDK2SA predicted the overall survival of stage I and II NSCLC patients (Figure 2). Sub-analysis of 134 patients with stage IA and IB disease showed that CDK1SA was an independent predictor only for recurrence, but the power of prediction was much better than conventional criteria such as tumor size and stage (Figure 3 and Table 5). The clinical relevance of CDK1SA as a marker for recurrence prediction is in agreement with the conclusion of the colon study [24]. In this colon study, CDK1SA was significantly elevated in microsatellite-stable tumors. Because most of colorectal cancers with stable microsatellites demonstrate chromosomal instability [35], CDK1SA may have value as a marker of genomic instability. Genomic instability has been reported to predict clinical outcomes in multiple cancer types, including lung cancer [36–39]; therefore, prediction of recurrence demonstrated in this study may reflect the genomic instability of the tumors. According to sub-analysis by histology, the predictive value of CDK1SA for recurrence was greater in adenocarcinoma than in SCC (Figure 4A and B). Since adenocarcinoma is the most common histologic type of colon cancer, there is a similarity in the role of CDK1SA in colon and lung adenocarcinoma. Interestingly, Cox regression analysis for recurrence revealed that CDK2SA expression was statistically significant in SCC but not in adenocarcinoma (Figure 4C, D and Table 6). This result suggests that CDK1 and CDK2 have different roles in adenocarcinoma and SCC.

In contrast to CDK1SA, CDK2SA predicted overall survival after surgery. This finding may be related to the chemo-sensitivity of the patients. Kaplan-Meier analysis indicated that the prognostic power of CDK2SA was significant in patients treated primarily with platinum-based chemotherapy after recurrence (Figure 5), suggesting that CDK2SA can predict platinum sensitivity/resistance. A similar trend was observed in our ovary study: tumors with high CDK2SA were more platinum-resistant in patients who underwent incomplete resection and subsequent platinum-based chemotherapy (unpublished data). The ability of cyclin E-associated kinase activity to predict the response to platinum-based chemotherapy in ovarian cancer patients was also reported by another group [21]. In addition, inhibition of CDK2, but not CDK1, induced growth arrest in lung cancer cell lines through anaphase catastrophe [40]. Taken together, these data indicate that CDK2 would be a good target for lung cancer treatment, and the measurement of CDK2SA could be useful for identifying patients who would receive the full benefit of CDK2 inhibitors.

A limitation of this study was that the number of cases in the sub-analysis for the outcome after platinum-based chemotherapy was low; however, the significant difference was quite clear (Figure 5). Prospective studies should be performed to clarify the predictive capacity of CDK2SA in platinum sensitivity/resistance in early-stage NSCLC patients after surgical resection.

In summary, this study suggested the possible clinical use of CDK1SA for recurrence prediction and CDK2SA for the prognosis of stage I and II NSCLC. Moreover, CDK2SA might be a predictor of platinum-based chemotherapy sensitivity/resistance. To the best of our knowledge, this is the first report that suggests a relationship between chemosensitivity and CDK activity in lung cancer. Thus, a combination of CDK1SA and CDK2SA might be helpful in decision-making regarding NSCLC treatment strategies.

## Conclusions

CDK1SA is a predictor of recurrence and CDK2SA is a predictor of overall survival in early-stage NSCLC after surgery.

## Electronic supplementary material

Additional file 1: **Distribution of lung tumors according to CDK1SA and CDK2SA.** Adenocarcinoma cases and SCC cases are plotted on a scatter diagram with logarithmic scales according to CDK1SA and CDK2SA. Black square; the specific activity was defined as 0.5 when the activity of CDK is lower than the detection limit of the assay. The detection limits for the activity of CDK1 and CDK2 are 10 and 2 maU/μL lysate, respectively. (PDF 293 KB)

## Abbreviations

NSCLC: Non-small cell lung cancer
SCC: Squamous cell carcinoma
CDKs: Cyclin-dependent kinases
CDK1SA: Specific activity of CDK1
CDK2SA: Specific activity of CDK2.

## References

[1] Reck M, Heigener DF, Mok T, Soria JC, Rabe KF (2013). Management of non-small-cell lung cancer: recent developments. Lancet.

[2] Williams DE, Pairolero PC, Davis CS, Bernatz PE, Payne WS, Taylor WF, Uhlenhopp MA, Fontana RS (1981). Survival of patients surgically treated for stage I lung cancer. J Thorac Cardiovasc Surg.

[3] Nesbitt JC, Putnam JB, Walsh GL, Roth JA, Mountain CF (1995). Survival in early-stage non-small cell lung cancer. Ann Thorac Surg.

[4] Spiro SG, Porter JC (2002). Lung cancer–where are we today? Current advances in staging and nonsurgical treatment. Am J Respir Crit Care Med.

[5] Martini N, Bains MS, Burt ME, Zakowski MF, McCormack P, Rusch VW, Ginsberg RJ (1995). Incidence of local recurrence and second primary tumors in resected stage I lung cancer. J Thorac Cardiovasc Surg.

[6] Kelsey CR, Marks LB, Hollis D, Hubbs JL, Ready NE, D'Amico TA, Boyd JA (2009). Local recurrence after surgery for early stage lung cancer: an 11-year experience with 975 patients. Cancer.

[7] Arriagada R, Bergman B, Dunant A, Le Chevalier T, Pignon JP, Vansteenkiste J, International Adjuvant Lung Cancer Trial Collaborative G (2004). Cisplatin-based adjuvant chemotherapy in patients with completely resected non-small-cell lung cancer. N Engl J Med.

[8] Douillard JY, Rosell R, De Lena M, Carpagnano F, Ramlau R, Gonzales-Larriba JL, Grodzki T, Pereira JR, Le Groumellec A, Lorusso V, Clary C, Torres AJ, Dahabreh J, Souquet PJ, Astudillo J, Fournel P, Artal-Cortes A, Jassem J, Koubkova L, His P, Riggi M, Hurteloup P (2006). Adjuvant vinorelbine plus cisplatin versus observation in patients with completely resected stage IB-IIIA non-small-cell lung cancer (Adjuvant Navelbine International Trialist Association [ANITA]): a randomised controlled trial. Lancet Oncol.

[9] Pisters KM, Evans WK, Azzoli CG, Kris MG, Smith CA, Desch CE, Somerfield MR, Brouwers MC, Darling G, Ellis PM, Gaspar LE, Pass HI, Spigel DR, Strawn JR, Ung YC, Shepherd FA, Cancer Care Ontario, American Society of Clinical Oncology (2007). Cancer Care Ontario and American Society of Clinical Oncology adjuvant chemotherapy and adjuvant radiation therapy for stages I-IIIA resectable non small-cell lung cancer guideline. J Clin Oncol.

[10] Strauss GM, Herndon JE, Maddaus MA, Johnstone DW, Johnson EA, Harpole DH, Gillenwater HH, Watson DM, Sugarbaker DJ, Schilsky RL, Vokes EE, Green MR (2008). Adjuvant paclitaxel plus carboplatin compared with observation in stage IB non-small-cell lung cancer: CALGB 9633 with the Cancer and Leukemia Group B, Radiation Therapy Oncology Group, and North Central Cancer Treatment Group Study Groups. J Clin Oncol.

[11] Kratz JR, He J, Van Den Eeden SK, Zhu ZH, Gao W, Pham PT, Mulvihill MS, Ziaei F, Zhang H, Su B, Zhi X, Quesenberry CP, Habel LA, Deng Q, Wang Z, Zhou J, Li H, Huang MC, Yeh CC, Segal MR, Ray MR, Jones KD, Raz DJ, Xu Z, Jahan TM, Berryman D, He B, Mann MJ, Jablons DM (2012). A practical molecular assay to predict survival in resected non-squamous, non-small-cell lung cancer: development and international validation studies. Lancet.

[12] Tomida S, Takeuchi T, Shimada Y, Arima C, Matsuo K, Mitsudomi T, Yatabe Y, Takahashi T (2009). Relapse-related molecular signature in lung adenocarcinomas identifies patients with dismal prognosis. J Clin Oncol.

[13] Malumbres M, Barbacid M (2009). Cell cycle, CDKs and cancer: a changing paradigm. Nat Rev Cancer.

[14] Begnami MD, Fregnani JH, Nonogaki S, Soares FA (2010). Evaluation of cell cycle protein expression in gastric cancer: cyclin B1 expression and its prognostic implication. Hum Pathol.

[15] Ishihara H, Yoshida T, Kawasaki Y, Kobayashi H, Yamasaki M, Nakayama S, Miki E, Shohmi K, Matsushima T, Tada S, Torikoshi Y, Morita M, Tamura S, Hino Y, Kamiyama J, Sowa Y, Tsuchihashi Y, Yamagishi H, Sakai T (2005). A new cancer diagnostic system based on a CDK profiling technology. Biochim Biophys Acta.

[16] Nakashima S, Natsugoe S, Matsumoto M, Kijima F, Takebayashi Y, Okumura H, Shimada M, Nakano S, Kusano C, Baba M, Takao S, Aikou T (2000). Expression of p53 and p21 is useful for the prediction of preoperative chemotherapeutic effects in esophageal carcinoma. Anticancer Res.

[17] Sjostrom J, Blomqvist C, Heikkila P, Boguslawski KV, Raisanen-Sokolowski A, Bengtsson NO, Mjaaland I, Malmstrom P, Ostenstadt B, Bergh J, Wist E, Valvere V, Saksela E (2000). Predictive value of p53, mdm-2, p21, and mib-1 for chemotherapy response in advanced breast cancer. Clin Cancer Res.

[18] Soria JC, Jang SJ, Khuri FR, Hassan K, Liu D, Hong WK, Mao L (2000). Overexpression of cyclin B1 in early-stage non-small cell lung cancer and its clinical implication. Cancer Res.

[19] Suzuki T, Urano T, Miki Y, Moriya T, Akahira J, Ishida T, Horie K, Inoue S, Sasano H (2007). Nuclear cyclin B1 in human breast carcinoma as a potent prognostic factor. Cancer Sci.

[20] Takano Y, Kato Y, van Diest PJ, Masuda M, Mitomi H, Okayasu I (2000). Cyclin D2 overexpression and lack of p27 correlate positively and cyclin E inversely with a poor prognosis in gastric cancer cases. Am J Pathol.

[21] Bedrosian I, Lee C, Tucker SL, Palla SL, Lu K, Keyomarsi K (2007). Cyclin E-associated kinase activity predicts response to platinum-based chemotherapy. Clin Cancer Res.

[22] Kim SJ, Nakayama S, Miyoshi Y, Taguchi T, Tamaki Y, Matsushima T, Torikoshi Y, Tanaka S, Yoshida T, Ishihara H, Noguchi S (2008). Determination of the specific activity of CDK1 and CDK2 as a novel prognostic indicator for early breast cancer. Ann Oncol.

[23] van Nes JG, Smit VT, Putter H, Kuppen PJ, Kim SJ, Daito M, Ding J, Shibayama M, Numada S, Gohda K, Matsushima T, Ishihara H, Noguchi S, van de Velde CJ (2009). Validation study of the prognostic value of cyclin-dependent kinase (CDK)-based risk in Caucasian breast cancer patients. Br J Cancer.

[24] Zeestraten EC, Maak M, Shibayama M, Schuster T, Nitsche U, Matsushima T, Nakayama S, Gohda K, Friess H, van de Velde CJ, Ishihara H, Rosenberg R, Kuppen PJ, Janssen KP (2012). Specific activity of cyclin-dependent kinase I is a new potential predictor of tumour recurrence in stage II colon cancer. Br J Cancer.

[25] Kim SJ, Nakayama S, Shimazu K, Tamaki Y, Akazawa K, Tsukamoto F, Torikoshi Y, Matsushima T, Shibayama M, Ishihara H, Noguchi S (2012). Recurrence risk score based on the specific activity of CDK1 and CDK2 predicts response to neoadjuvant paclitaxel followed by 5-fluorouracil, epirubicin and cyclophosphamide in breast cancers. Ann Oncol.

[26] Dobashi Y, Jiang SX, Shoji M, Morinaga S, Kameya T (2003). Diversity in expression and prognostic significance of G1/S cyclins in human primary lung carcinomas. J Pathol.

[27] Esposito V, Baldi A, Tonini G, Vincenzi B, Santini M, Ambrogi V, Mineo TC, Persichetti P, Liuzzi G, Montesarchio V, Wolner E, Baldi F, Groeger AM (2004). Analysis of cell cycle regulator proteins in non-small cell lung cancer. J Clin Pathol.

[28] Hayashi H, Ogawa N, Ishiwa N, Yazawa T, Inayama Y, Ito T, Kitamura H (2001). High cyclin E and low p27/Kip1 expressions are potentially poor prognostic factors in lung adenocarcinoma patients. Lung Cancer.

[29] Jin M, Inoue S, Umemura T, Moriya J, Arakawa M, Nagashima K, Kato H (2001). Cyclin D1, p16 and retinoblastoma gene product expression as a predictor for prognosis in non-small cell lung cancer at stages I and II. Lung Cancer.

[30] Morero JL, Poleri C, Martin C, Van Kooten M, Chacon R, Rosenberg M (2007). Influence of apoptosis and cell cycle regulator proteins on chemotherapy response and survival in stage IIIA/IIIB NSCLC patients. J Thorac Oncol.

[31] Yoshida T, Tanaka S, Mogi A, Shitara Y, Kuwano H (2004). The clinical significance of Cyclin B1 and Wee1 expression in non-small-cell lung cancer. Ann Oncol.

[32] Postel-Vinay S, Vanhecke E, Olaussen KA, Lord CJ, Ashworth A, Soria JC (2012). The potential of exploiting DNA-repair defects for optimizing lung cancer treatment. Nat Rev Clin Oncol.

[33] Singhal S, Vachani A, Antin-Ozerkis D, Kaiser LR, Albelda SM (2005). Prognostic implications of cell cycle, apoptosis, and angiogenesis biomarkers in non-small cell lung cancer: a review. Clin Cancer Res.

[34] Schwartz GK, Shah MA (2005). Targeting the cell cycle: a new approach to cancer therapy. J Clin Oncol.

[35] Walther A, Johnstone E, Swanton C, Midgley R, Tomlinson I, Kerr D (2009). Genetic prognostic and predictive markers in colorectal cancer. Nat Rev Cancer.

[36] Albertson DG, Collins C, McCormick F, Gray JW (2003). Chromosome aberrations in solid tumors. Nat Genet.

[37] Carter SL, Eklund AC, Kohane IS, Harris LN, Szallasi Z (2006). A signature of chromosomal instability inferred from gene expression profiles predicts clinical outcome in multiple human cancers. Nat Genet.

[38] Mettu RK, Wan YW, Habermann JK, Ried T, Guo NL (2010). A 12-gene genomic instability signature predicts clinical outcomes in multiple cancer types. Int J Biol Markers.

[39] Nakamura H, Saji H, Idiris A, Kawasaki N, Hosaka M, Ogata A, Saijo T, Kato H (2003). Chromosomal instability detected by fluorescence in situ hybridization in surgical specimens of non-small cell lung cancer is associated with poor survival. Clin Cancer Res.

[40] Galimberti F, Thompson SL, Liu X, Li H, Memoli V, Green SR, DiRenzo J, Greninger P, Sharma SV, Settleman J, Compton DA, Dmitrovsky E (2010). Targeting the cyclin E-Cdk-2 complex represses lung cancer growth by triggering anaphase catastrophe. Clin Cancer Res.

[CR41] The pre-publication history for this paper can be accessed here:http://www.biomedcentral.com/1471-2407/14/755/prepub

