# Supplementary figures and images for: Cyclin-dependent kinase-specific activity predicts the prognosis of stage I and stage II non-small cell lung cancer
Source: BMC Cancer. 2014 Oct 9;14:755. doi: 10.1186/1471-2407-14-755 (PMC4198674; doi:10.1186/1471-2407-14-755)

**A. Adenocarcinoma (n = 118)**

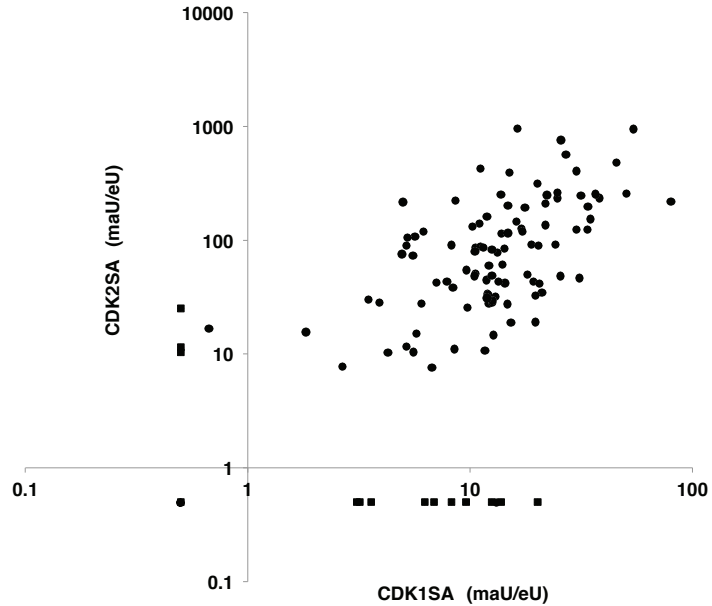

**B. SCC (n = 53)**

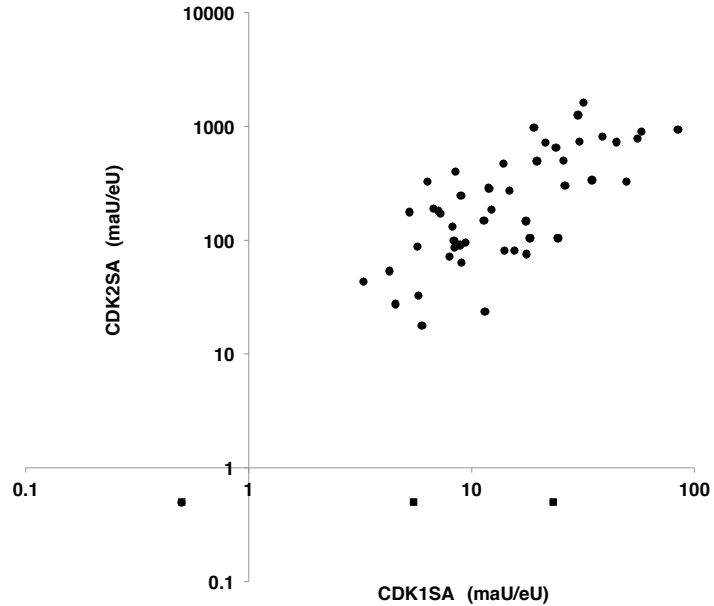

Supplement: Supplementary file 1 — Additional file 1: Distribution of lung tumors according to CDK1SA and CDK2SA. Adenocarcinoma cases and SCC cases are plotted on a scatter diagram with logarithmic scales according to CDK1SA and CDK2SA. Black square; the specific activity was defined as 0.5 when the activity of CDK is lower than the detection limit of the assay. The detection limits for the activity of CDK1 and CDK2 are 10 and 2 maU/μL lysate, respectively. (PDF 293 KB) [file 12885_2014_4934_MOESM1_ESM.pdf]
